# DNA@Mn_3_(PO_4_)_2_ Nanoparticles Supported with Graphene Oxide as Photoelectrodes for Photoeletrocatalysis

**DOI:** 10.1186/s11671-016-1784-z

**Published:** 2017-01-06

**Authors:** Lixia Gao, Jiale Xie, Xiaoqing Ma, Man Li, Ling Yu

**Affiliations:** 1 Institute for Clean energy & Advanced Materials, Faculty of Materials & Energy, Southwest University, Chongqing, 400715 China; 2 Chongqing Key Laboratory for Advanced Materials and Technologies of Clean Energies, Chongqing, 400715 China; 3 Institute of Materials Science and Devices, Suzhou University of Science and Technology, Suzhou, 215011 China

**Keywords:** Photoelectrocatalysis, Photoelectrode, DNA, Mn_3_(PO_4_)_2_, Nanoparticle

## Abstract

A novel deoxyribose nucleic acid (DNA)-based photoelectrode consisting of DNA@Mn_3_(PO_4_)_2_ nanoparticles on graphene oxide (GO) sheets was successfully fabricated for photoelectrocatalysis. DNA served as a soft template to guide the nucleation and growth of Mn_3_(PO_4_)_2_ nanoparticles in the synthesis of Mn_3_(PO_4_)_2_ nanoparticles. More importantly, the DNA also serves as semiconductor materials to adjust charge transport. Under UV light irradiation (180–420 nm, 15 mW/cm^2^), the photocurrent density of DNA@ Mn_3_(PO_4_)_2_/GO electrodes reached 9 μA/cm^2^ at 0.7 V bias (vs. SCE). An applied bias photon-to-current efficiency (ABPE) of ~0.18% can be achieved, which was much higher than that of other control electrodes (<0.04%). In this DNA-based photoelectrode, well-matched energy levels can efficiently improve charge transfer and reduce the recombination of photogenerated electron-hole pairs.

**Electronic supplementary material:**

The online version of this article (doi:10.1186/s11671-016-1784-z) contains supplementary material, which is available to authorized users.

## Background

Energy crisis and environmental issue at global level are important topics and force us to search renewable clean energy [1]. Sunlight is an inexpensive, non-polluting, abundant, and endlessly renewable source of clean energy. Among the technologies for converting sunlight, photoelectrocatalysis combined the concepts of photovoltaics and wet-chemical photosynthesis has the ability to split water through solar energy to produce hydrogen [2]. Normally, broadly used photoelectrode materials are inorganic semiconductor material, such as metal oxides, silicates, and molybdates [3–9]. Phosphate can accelerate photoelectrochemical reactions and Li et al*.* reported cobalt phosphate as an oxygen evolution co-catalyst and used as a potential photoelectrocatalyst for efficient solar water splitting [10, 11]. Manganese phosphate, (Mn_3_(PO_4_)_2_, as one of phosphates has been used as cathode material for lithium ion battery [12], a water oxidation catalyst [13] and sensing materials [14]. As far as we know, no phosphate was studied as photoelectrocatalyst.

The method of solid-phase synthesis is commonly used to prepare Mn_3_(PO_4_)_2_. Therefore, the size of synthesized Mn_3_(PO_4_)_2_ nanoparticles is in the range of several micrometers. Deoxyribose nucleic acid (DNA), a kind of nucleic acid carrying the genetic information of living organisms, shows to be promising in preparing materials with novel structures and functions [15]. Tremendous attention has been paid because of its efficiency and versatility in controlling of nanostructure growth. The programmable nature and rich molecular interaction with a variety of species enable DNA as a template to guide and support functional nanomaterials synthesis [16–19]. To reduce the size and increase the specific surface area, we use DNA molecules as a soft template for assisting crystal nucleation and uniformly growth of Mn_3_(PO_4_)_2_. Moreover, as a p-type semiconductor, DNA can serve as partial electrode material for adjusting charge transportation [20–22].

Limited charge transport at the interface between nanomaterial and electrode surface is a critical issue in photoelectrocatalysis. Two-dimensional nanomaterial can provide the opportunity to functionalize this interface. As a star nanomaterial, graphene oxide (GO) was obtained by the oxidation of graphite powders and has a p-type semiconductor characteristics [8, 23], resulting from oxygen’s higher electronegativity than carbon atoms [24]. Besides, DNA and GO can bind together by strong interactions, such as van der Waals, *π*-*π* stacking, and combination of hydrogen [25].

Herein, we fabricated a novel DNA-based photoelectrode consisted with DNA@ Mn_3_(PO_4_)_2_ nanoparticles on GO sheets. The DNA as a soft template can assist the nucleation and growth of Mn_3_(PO_4_)_2_ nanoparticles. GO as the electron-blocking layer can reduce charge recombination, which can also enhance the light absorption partially. Under UV light irradiation (*λ*: 180–420 nm, 15 mW/cm^2^), the photocurrent density of DNA@ Mn_3_(PO_4_)_2_/GO electrodes can reach up to 9 μA/cm^2^ at 0.7 V bias (vs. SCE). An ABPE efficiency of ~0.18% can be achieved, which is much higher than that of other control electrodes (<0.04%). In this DNA-based photoelectrode, well-matched energy levels can efficiently improve charge transfer and reduce the recombination of photogenerated electron-hole pairs.

## Methods

### Reagents

Single-stranded deoxyribonucleic acid (ss-DNA), double-strand DNA (ds-DNA), low molecular weight DNA (lm-DNA), MnO_2_, K_3_PO_4_, and phosphate-buffered saline (PBS) are all purchased from Sigma-Aldrich (China). Graphite with purity higher than 98.3% was obtained from Sinocarbon Materials Technology Co., Ltd., China. Experimental used water is deionized water ((DI H_2_O), 18.2 MΩ/cm, ELGA Lab Water, England).

### Synthesis of Graphite Oxide Nanosheets and DNA@Mn_3_(PO4)_2_ Nanocomposite

#### Graphite Oxide (GO) Nanosheets Synthesis

GO nanosheets were prepared from graphite powders by a modified Hummers method [26, 27]. Firstly, 0.5 g graphite, 0.5 g NaNO_3_, and 23 mL H_2_SO_4_ (98%) were together stirred in an ice bath, then slowly mixed with 3 g of KMnO4. The mixture was placed in a water bath at 35 ± 5 °C stirring for about 1 h until the paste appears. Secondly, 100 ml DI H_2_O was added into the flask to dilute the mixture. Thirdly, 3 ml H_2_O_2_ (30%) was drop-casted into the reaction mixture. Fourthly, product was harvested through centrifugation and thoroughly washed by HCl (5%, *v*/*v*) and DI H_2_O. GO (6 mg/mL) was dispersed use ultrasonic about 6 h with an ultrasonic power of 65 W. Finally, the homogeneous solution was centrifuged about 10 min to collect the supernatant for reserve.

#### DNA@Mn_3_(PO_4_)_2_ Nanocomposite Synthesis

The DNA@Mn_3_(PO_4_)_2_ was prepared according to literature [14]. In brief, firstly, 0.1 ml DNA (10 mg/ml) was dissolved into 0.9 ml DI H_2_O and boiled for 10 min then ice cooling to prevent forming of double strand helix. Secondly, 1 ml DNA solution, 1 ml MnSO_4_ (0.1 M), and 9 ml DI H_2_O were mixed under stirring for 10 min. Thirdly, 1 ml K_3_PO_4_ (0.1 M) and 9 ml H_2_O were stirred for 1 h at 60 °C water bath. Then DNA, MnSO_4_, and K_3_PO_4_ mixture was stirred for another 1 h until the solution became transparent which indicates Mn^2+^ ions and phosphate groups of DNA reacted completely. The obtained solution was centrifuged at 9000 r/min for 10 min, and the DNA@ Mn_3_(PO_4_)_2_ pellet was collected. The supernatant was tested by UV-vis measurement at the range of 200–500 nm wavelength. Finally, the synthesized DNA@ Mn_3_(PO_4_)_2_ nanocomposite was dispersed in DI H_2_O.

### Fabrication of DNA@Mn_3_(PO_4_)_2_/GO Electrode

Fluorine-doped tin oxide (FTO) coated glass (2.5 cm × 1 cm, sheet resistance <10 Ω/sq.) as a work electrode was ultrasonically pre-cleaned with acetone and ethanol for 30 min and dried with nitrogen stream. Subsequently, oxygen plasma (Mycro Technologies Co., Ltd.) was used to clear the FTO surface for 5 min. Then 6 mg/ml GO homogenous solution was spin coating onto pre-cleaned FTO surface and dried in room temperature. Finally, DNA@Mn_3_(PO_4_)_2_ solution was further drop-casted on GO/FTO surface.

### Characterization of Materials and Electrodes

Structural properties of synthesized nanocomposite were measured using JSM-6510LV scanning electron microscopy (SEM). JEM-2100 transmission electron microscopy (TEM) was further used to characterize the morphology in detail. GO and DNA were characterized by atomic force microscopy (AFM). P, N, O, and Mn elements were identified by X-ray photoelectron spectroscopy (XPS). FTIR transmittance spectra were obtained from NICOLET 6700. UV-2550 spectrophotometer recorded UV-vis absorption spectra, and the background contribution of the FTO glass to the absorption spectra was eliminated. The electrode of DNA@Mn_3_(PO_4_)_2_/GO was examined by energy dispersive X-ray spectroscopy (EDS, equipment with JSM-6510 LV, Japan).

### Photoelectrochemical Measurements

Photoelectrochemical measurements were carried out on an electrochemical workstation (CHI 760E, Chen Hua Instruments Co. Ltd.). A three-electrode system was employed with a FTO as a working electrode (photoelectrode), an Hg/HgCl_2_/KCL electrode (saturated calomel reference electrode, SCE) as a reference electrode, and a 2.5 cm^2^ platinum plate as a counter electrode. All tests were conducted with a self-built 2 cm × 2 cm × 1 cm quartz electrolytic cell in 0.01 M phosphate-buffered saline (PBS). Ultra violet light Source (MUA-165, Japan) was used to irradiate the work electrode under UV light (*λ*: 180–420 nm, 15 mW/cm^2^). The experimental measurements were carried out at room temperature without seal. Linear sweep voltammetry (LSV) was recorded at a scan rate of 50 mV/s. The amperometric *i*–*t* curve was recorded to measure photocurrent response under open circuit potential-time with intermittent light irradiation.

## Results

### Unique Morphology and Properties of DNA@Mn_3_(PO_4_)_2_/GO

**Scheme 1:**
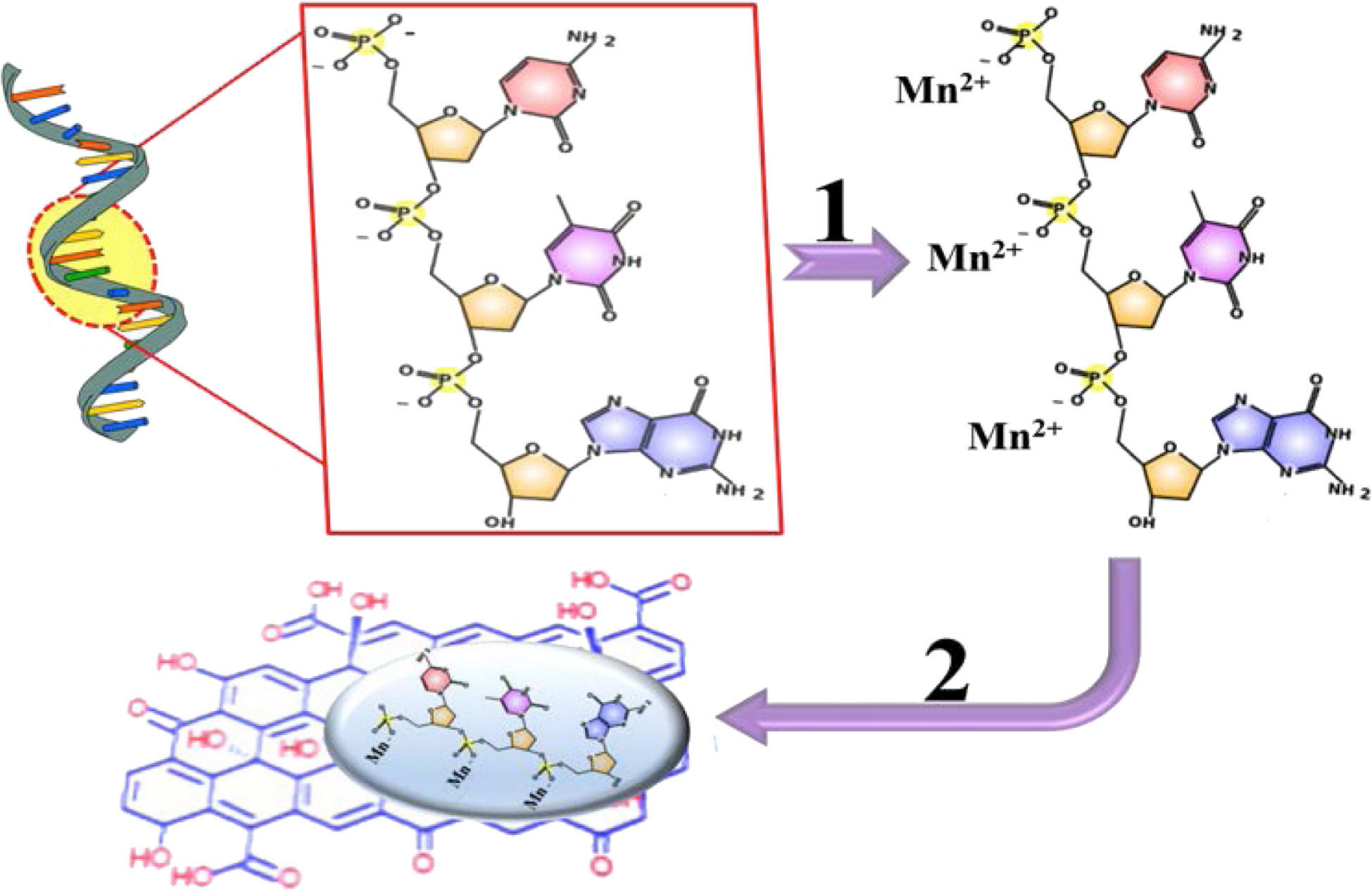
Schematic synthesis of DNA@Mn_3_(PO_4_)_2_/GO. (1) The phosphate groups of ssDNA react with Mn^2+^ ions and form Mn_3_(PO_4_)_2_ nanoparticles. (2) DNA@Mn_3_(PO_4_)_2_ is assembled onto GO nanosheets

The synthesis processes of DNA@ Mn_3_(PO_4_)_2_ nanocomposite and DNA@ Mn_3_(PO_4_)_2_/GO electrode are schematically shown in Scheme 1. The DNA was used to guide the crystals nucleation of Mn_3_(PO_4_)_2_ nanoparticles. In the presence of Mn^2+^ solution, the rich negatively charged PO_4_^3−^ groups regularly arranged on the sugar-phosphate backbone of DNA attracted Mn^2+^ ions onto the surface of DNA molecule strains. The following growth of Mn_3_(PO_4_)_2_ was promoted by potassium phosphate (K_3_PO_4_). Due to the low solubility of Mn_3_(PO_4_)_2_, the nanoparticles precipitated in the solution. Assembling of DNA@ Mn_3_(PO_4_)_2_ nanoparticles onto GO nanosheets was achieved by *π*-*π* non-covalent conjugation and electrostatic interaction between the base pairs of DNA and the functionalized surface of GO nanosheets.

**Fig. 1:**
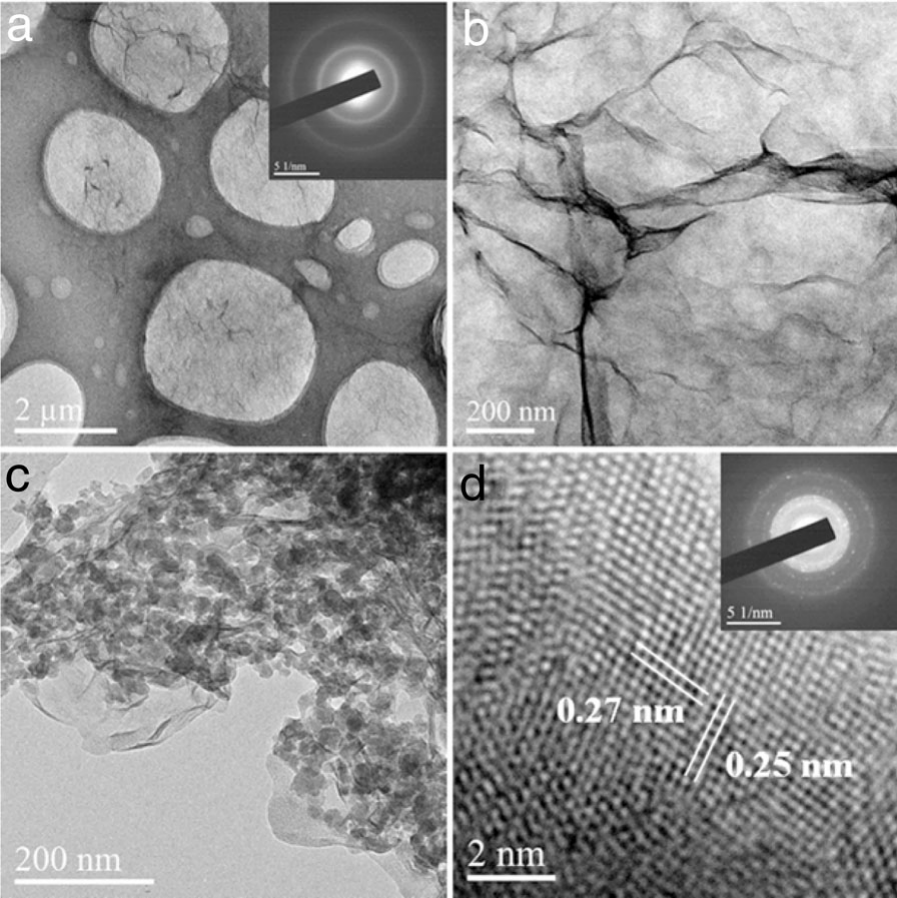
Classical structure analysis by SEM (Additional file 1: Figure S2) and TEM. **a**, **b** Low-magnification and high-magnification TEM images of GO sheets. Inset of Fig. 1a is the SAED pattern of GO sheets. **c** Low-magnification TEM image of DNA@Mn_3_(PO_4_)_2_/GO. **d** HRTEM image of Mn_3_(PO_4_)_2_ nanoparticle. Inset of Fig. 1d is the SAED pattern of DNA@Mn_3_(PO_4_)_2_/GO

**Fig. 2:**
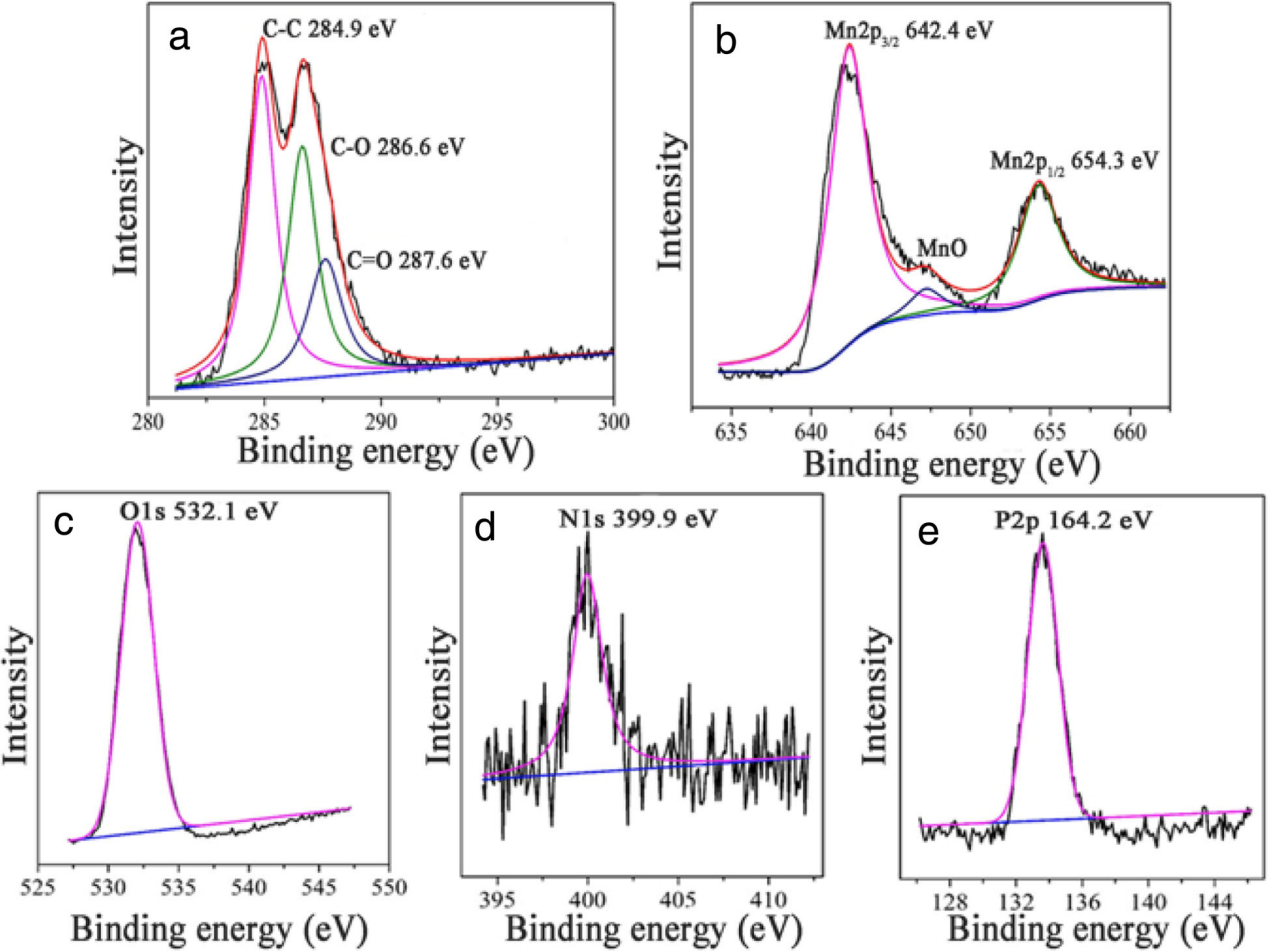
High-resolution XPS analysis of DNA@Mn_3_(PO_4_)_2_/GO. **a** C1s, **b** Mn2p, **c** O1s, **d** N1s, and **e** P2p

TEM images of GO nanosheets are shown in Fig. 1a, b. The selected area electron diffraction (SAED) pattern of GO is shown as the inset of Fig. 1a. The well-defined diffraction cycles confirm the integrity of GO sheets. The GO thickness is in nanometer scale (Additional file 1: Figure S1A), as thin as the yarn, and there are some wrinkles covering the electrode surface. After the drop-coating of DNA@Mn_3_(PO_4_)_2_, the DNA@Mn_3_(PO_4_)_2_ nanoparticles are dispersed onto the surface of GO sheets uniformly (Fig. 1c). The DNA@ Mn_3_(PO_4_)_2_ nanoparticle size is about 20–50 nm. HRTEM image shows regular lattice fringes, indicating Mn_3_(PO_4_)_2_ nanoparticle has relative good crystal quality (Fig. 1d). The fringe spacing as shown in Fig. 1d is 0.27 and 0.25 nm. The SAED pattern are well-consistent with the diffraction peaks of 32.90° and 35.59° in standard PDF-card of Mn_3_(PO_4_)_2_ (card number: 33–0901) and experimental XRD pattern of Mn_3_(PO_4_)_2_ as shown in Additional file 1: Figure S2. As shown in the inset of Fig. 2d, some light ring consisted of diffraction spots can be distinguished, so Mn_3_(PO_4_)_2_ nanoparticles were well-grown under 60 °C water bath.

The DNA@Mn_3_(PO_4_)_2_/GO sample composition analysis of C 1s, Mn 2p, O 1s, N 1s, and P 2p peaks was conducted with XPS. The C 1s peak (Fig. 2a) can be divided into three most prominent peaks using Gauss fitting. The peaks of 284.9, 286.6, and 287.6 eV can be attributed to C=C, C–O, and C=O, respectively. This indicates that the GO nanosheets have been modified to the electrode surface. The Mn 2p XPS (Fig. 2b) shows two important peaks with a binding energy of 642.4 and 654.3 eV, corresponding to the Mn 2p3/2 and Mn 2p1/2 of Mn_3_(PO_4_)_2_. Another 647.5 eV peak comes from MnO, indicating the existence of MnO doping in DNA@ Mn_3_(PO_4_)_2_. The O 1s peak (Fig. 2c) appearing at binding energy of 532.1 eV is originally from the phosphate groups in DNA@Mn_3_(PO_4_)_2_/GO. The N 1s peak (Fig. 2d) can be identified at 399.9 eV, directly demonstrating the existence of DNA molecules in hybrid electrodes. The peak of 133.5 eV can be attributed to P 2p (Fig. 2e) of phosphate. The XPS spectra of N 1s and P 2p peaks can fully demonstrate DNA adsorbed on the surface of the electrode [28]. This indicates that Mn^2+^ ion has combined with phosphate radical of DNA and it plays a key role for material synthesis.

**Fig. 3:**
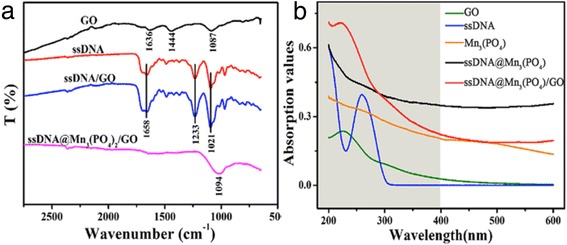
**a** FTIR transmittance spectra of GO, ssDNA, ssDNA/GO, and ssDNA@Mn_3_(PO_4_)_2_/GO. **b** UV-vis absorption spectra of GO, ssDNA, Mn_3_(PO_4_)_2_, ssDNA@Mn_3_(PO_4_)_2_, and ssDNA@Mn_3_(PO_4_)_2_/GO in 0.01 M PBS solution. The *shadow area* is the range of UV light used during measurements

FTIR was used to further characterize the DNA@Mn_3_(PO_4_)_2_/GO (Fig. 3a). The prominent characteristic peaks at about 3200 cm^−1^ (O–H stretching vibrations), 1636 cm^−1^ (C=O stretching vibrations), and the bands at 1444 cm^−1^ (C–O stretching vibrations of carbonyl and carboxylic groups) and 1087 cm^−1^ (C–O stretching vibrations) are derived from GO sheets [29]. Intense bands at about 1657 cm^−1^ (C=O stretching vibrations corresponding to the double bonds of DNA) and 1093 cm^−1^, 1233 cm^−1^ (PO_2_ antisymmetric and symmetric stretching vibrations attributable to the DNA strand) are corresponding to DNA molecules. The new band near 3300 cm^−1^ is the stretching vibrations of O–H, N–H bonds from the deoxyribose of DNA molecules indicating the successful of incorporating DNA on GO sheets. DNA@ Mn_3_(PO_4_)_2_/GO nanocomposite in the vicinity of 3200 cm^−1^ (O–H stretching vibrations), 1058 and 1021 cm^−1^ showed absorption peaks was obtained from C–O and PO_2_ antisymmetric stretch.

The optical absorption properties of different electrodes were revealed by UV-vis absorption spectra in 200–600 nm (Fig. 3b). Two features of GO absorption plot (green line) can be used to identify GO: a maximum at 231 nm, which denotes *π*-π* transition peaks of aromatic C–C bonds, and a gentle bump at about 300 nm [30] was corresponding to *n*-π* transition shoulder peaks of C = O bond [29]. The two peaks have certain red shift by conjugation. The ssDNA (blue line) electrode has a strong feature absorption peak at 260 nm [31]. Whereas Mn_3_(PO_4_)_2_ has a weak absorption peak near 260 nm, indicating a wide band gap of Mn_3_(PO_4_)_2_. UV-vis absorption spectra of ssDNA@Mn_3_(PO_4_)_2_ shows lower absorbance peaks than DNA, suggesting the phosphoric acid groups on ssDNA reacted with Mn^2+^ ions. While a peak at ~231 nm of DNA@Mn_3_(PO_4_)_2_/GO is further observed, attributing to the *π*-π* accumulation of GO and DNA. Furthermore, the amount of DNA in the DNA@Mn_3_(PO_4_)_2_ composite was quantified by comparing the absorbance value at 260 nm of the DNA solution before and after forming Mn_3_(PO_4_)_2_ precipitates. As shown in Additional file 1: Figure S3A, after reaction with MnSO4 and K_3_PO_4_, the absorbance value of the solution decreased. Based on the DNA calibration curve (Additional file 1: Figure S3B), the decreased DNA quality is 6.6 μg.

### Photoelectrochemical Behaviors of DNA@ Mn_3_(PO_4_)_2_/GO

**Fig. 4:**
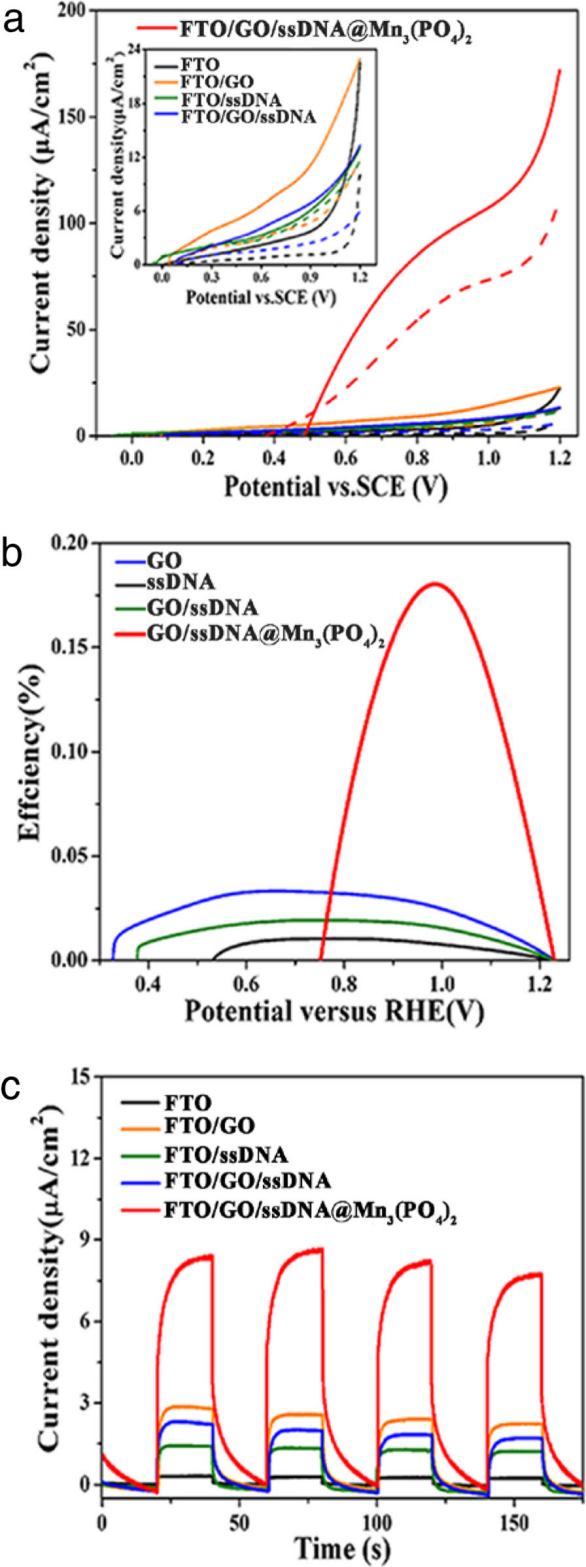
Photoelectrochemical measurements. **a** LSV plots of different electrodes under UV light and without UV light (*λ*: 180–420 nm, 15 mW/cm^2^). **b** Applied bias hydrogen conversion efficiency calculated from LSV plots. **c** Photocurrent response at 0.7 V (vs*.* SCE) under UV light irradiation

Figure 4a, c shows that the LSV and *i*–*t* curves of FTO, FTO/GO, FTO/ssDNA, FTO/GO/ssDNA, and FTO/GO/ssDNA@ Mn_3_(PO_4_)_2_ electrodes under UV light irradiation. The photocurrent density of GO/ssDNA@ Mn_3_(PO_4_)_2_ is obviously higher than other electrodes. However, the dark current density is also higher, indicating the corrosion maybe happen on this electrode. This should be detail investigated in further research. As shown in Fig. 4b, the GO/ssDNA@Mn_3_(PO_4_)_2_ electrode can deliver applied bias photon-to-current efficiency of ~0.18%, while GO, ssDNA, and GO/ssDNA electrodes only achieved 0.03, 0.01, and 0.019%, respectively. Photocurrent responses from all electrodes indicate that a transient photocurrent increase after UV irradiation. Furthermore, no visible charge recombination was observed from the photoelectrochemical measurements.

The photoelectrochemical behaviors were investigated in 0.01 M PBS (pH = 7.4) with three-electrode configuration (Additional file 1: Figure S4). The LSV and amperometric *i*–*t* techniques were used to analysis electrode photoelectrochemical behaviors. Additional file 1: Figure S4a and b shows the light responses with various DNA molecules. The photocurrent densities of dsDNA and lmDNA are 6 and 5 μA/cm^2^, respectively, whereas the photocurrent density of ssDNA can reach up to 9 μA/cm^2^. Therefore, the ssDNA can deliver higher photoelectric response under UV light irradiation. Furthermore, we optimized the GO concentration and the results are shown in Additional file 1: Figure S4c and 4d. 6 mg/ml GO solution can obtain current density of 9 μA/cm^2^, whereas 3 and 10 mg/ml can only reach 5 and 3 μA/cm^2^, respectively. GO is a p-type semiconductor, therefore only a suitable GO concentration (6 mg/ml) could obtain good electrical behaviors and good photocurrent response. Mn^2+^ ions concentration is another important factor, which can influence the performance of electrodes. As shown in Additional file 1: Figure S4e and 4f, the photocurrent density of 0.1 M Mn^2+^ ions can reach up to 9 μA/cm^2^, but 0.05 and 0.2 M Mn^2+^ ions only show 5 and 2 μA/cm^2^. This maybe indicates 0.1 M Mn^2+^ ions are completely combined with phosphate of DNA strand.

### Photoelectrochemical Mechanism of DNA@ Mn_3_(PO_4_)_2_/GO

**Fig. 5:**
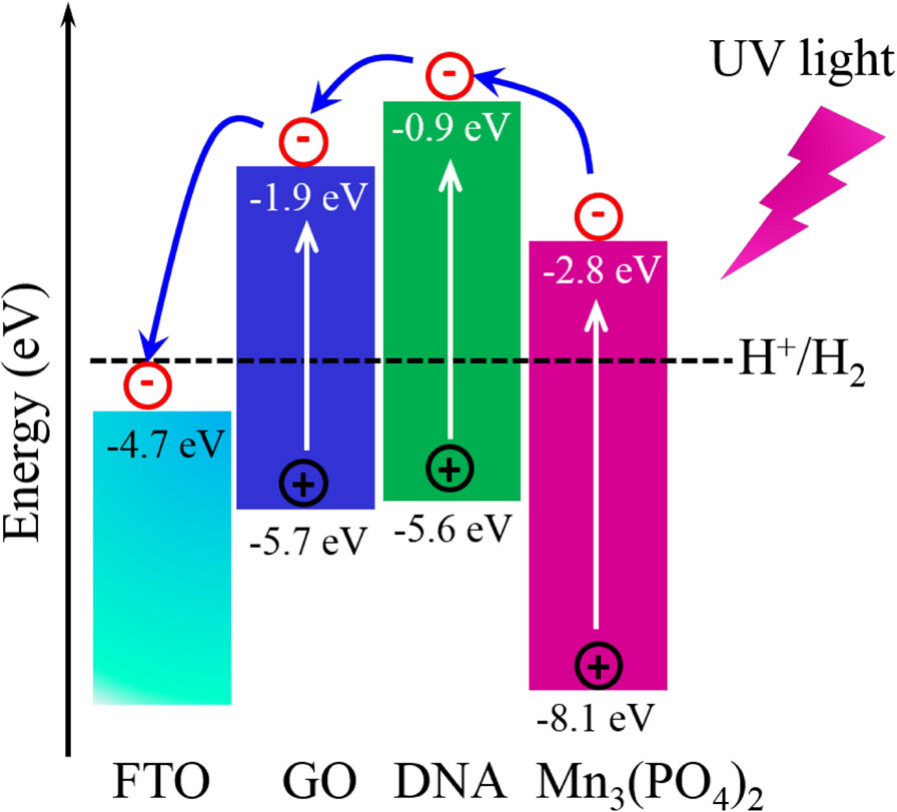
Energy-level diagram of DNA@Mn_3_(PO_4_)_2_/GO electrode

The energy level diagram of DNA@Mn_3_(PO_4_)_2_/GO electrode is shown in Fig. 5. The band edge of Mn_3_(PO_4_)_2_ was estimated from the UV-vis light absorption spectroscopy and cyclic voltammetry (CV) curve of Mn_3_(PO_4_)_2_ powder (Additional file 1: Figure S5). Under UV light irradiation, the electron-hole pairs can be generated in GO, DNA, and Mn_3_(PO_4_)_2_, resulting from the high energy of UV light (2.95–6.89 eV). The excited electrons in Mn_3_(PO_4_)_2_ can be further excited and transferred to DNA. And then, the excited electrons can transfer as the gradient of energy level. At last, the electrons collected through FTO electrode were used to catalyze the water splitting on Pt counter electrode. Meanwhile, the photogenerated holes are easy to transfer/transport through GO and DNA due to their p-type properties. Then the holes can oxidize Mn^2+^ to Mn^3+^ or/and Mn^4+^, and thus promote the water oxidation on the surface of Mn_3_(PO_4_)_2_ nanoparticles.

## Discussion

To investigate the enhanced photoelectric conversion efficiency of nanoparticle photoelectrodes in this work, we firstly fabricated the DNA-based nanoparticle photoelectrode (DNA@Mn_3_(PO_4_)_2_/GO) for photoelectrochemical solar cells (PECs). DNA was often used as a template to guide the nucleation and growth of nanoparticle materials [14]. DNA and GO can bind together by strong interactions including van der Waals, *π*-*π* stacking and combination of hydrogen. Therefore, we used the DNA as a soft template to guide the synthesis of Mn_3_(PO_4_)_2_ nanoparticles with the size in nanoscales. Recent reports revealed that the Mn-based oxide catalyst could deliver a significant OER electrocatalytic performance [32]. GO sheets with the p-type semiconductor characteristic can efficiently reduce the recombination, while Mn_3_(PO_4_)_2_ can harvest the UV light largely. DNA@Mn_3_(PO_4_)_2_/GO electrodes provide well-matched energy levels to improve the photoelectron transfer from the semiconducting film to the electrode and reduce the recombination of photoelectrons and holes.

In order to further enhance the photoelectric conversion efficiency, we optimized the molecular weight of the DNA, the GO concentration, Mn ion concentration. Under UV light irradiation (180–420 nm, 15 mW/cm^2^), the photocurrent density of DNA@Mn_3_(PO_4_)_2_ Mn_3_(PO_4_)_2_/GO electrodes reached 9 μA/cm^2^ at 0.7 V bias (vs. SCE). An ABPE efficiency of ~0.18% can be achieved—much higher than that of other control electrodes (<0.04%). Thus, we believe our results represent a potential application in enhancing the photoelectric conversion efficiency of nanomaterials.

## Conclusions

In summary, we have developed a novel DNA@Mn_3_(PO_4_)_2_/GO photoelectrode supported with GO sheets. The DNA@Mn_3_(PO_4_)_2_/GO electrodes possess sensitive and higher response to ultraviolet light. Under UV light irradiation (*λ*: 180–420 nm, 15 mW/cm^2^), the photocurrent intensity of DNA@Mn_3_(PO_4_)_2_/GO electrodes can reach up to 9 μA/cm^2^ at 0.7 V bias (vs. SCE), corresponding to applied bias photon-to-current efficiency of 0.18%, which is higher than other different electrodes prepared for comparison. DNA@Mn_3_(PO_4_)_2_/GO electrodes provide a well-matched energy level to improve the photoelectron transfer rate from the semiconducting film to the electrode and reduce the recombination of photoelectrons and holes. This work demonstrates that DNA behaved as a useful biomaterial for the synthesis of a photoelectroactive hybrid film with improved performance.

## Additional File

Additional file 1: Figure S1. AFM images of (A) GO drop-casted on freshly cleaved mica surface, (B) DNA drop-casted on freshly cleaved mica surface. **Figure S2**. XRD pattern of Mn_3_(PO_4_)_2_ powders. A. Mn_3_(PO_4_)_2_; B. Standard XRD pattern of Mn_3_(PO_4_)_2_ from the PDF card of 33–0901.** Figure S3**. Absorbance of DNA solution before and after react with MnSO4 and K3PO4 (A); concentration vs. absorbance calibration of DNA solution (B). **Figure S4**. Linear sweep voltammetry curves of (a) ssDNA, dsDNA, and lmDNA synthesized nanocomposite (c) different concentrations of GO synthesized nanocomposite (e) different concentration of Mn_3_(PO_4_)_2_; and photocurrent response of (b) ssDNA, dsDNA, and lmDNA synthesized nanocomposite (d) different concentrations of GO synthesized nanocomposite (f) different concentrations of Mn_3_(PO_4_)_2_. **Figure S5**. (a) UV-vis diffuses reflectance spectra of Mn_3_(PO_4_)_2_ powder, (b) first derivative absorption spectra of Mn_3_(PO_4_)_2_ powder. (c) Cyclic voltammetry (CV) curve of Mn_3_(PO_4_)_2_ on glassy carbon electrode in 0.1 M KCl solution. (DOCX 2269 kb)
