# Supplementary material for: DNA@Mn3(PO4)2 Nanoparticles Supported with Graphene Oxide as Photoelectrodes for Photoeletrocatalysis
Source: Nanoscale Res Lett. 2017 Jan 6;12:17. doi: 10.1186/s11671-016-1784-z (PMC5216005; doi:10.1186/s11671-016-1784-z)
Supplement: Additional file 1: Figure S1. — AFM images of (A) GO drop-casted on freshly cleaved mica surface, (B) DNA drop-casted on freshly cleaved mica surface. Figure S2. XRD pattern of Mn3(PO4)2 powders. A. Mn3(PO4)2; B. Standard XRD pattern of Mn3(PO4)2 from the PDF card of 33–0901. Figure S3. Absorbance of DNA solution before and after react with MnSO4 and K3PO4 (A); concentration vs. absorbance calibration of DNA solution (B). Figure S4. Linear sweep voltammetry curves of (a) ssDNA, dsDNA, and lmDNA synthesized nanocomposite (c) different concentrations of GO synthesized nanocomposite (e) different concentration of Mn3(PO4)2; and photocurrent response of (b) ssDNA, dsDNA, and lmDNA synthesized nanocomposite (d) different concentrations of GO synthesized nanocomposite (f) different concentrations of Mn3(PO4)2. Figure S5. (a) UV-vis diffuses reflectance spectra of Mn3(PO4)2 powder, (b) first derivative absorption spectra of Mn3(PO4)2 powder. (c) Cyclic voltammetry (CV) curve of Mn3(PO4)2 on glassy carbon electrode in 0.1 M KCl solution. (DOCX 2269 kb) [file 11671_2016_1784_MOESM1_ESM.docx]

DNA@Mn_3_(PO_4_)_2_ Nanoparticles Supported with Graphene Oxide as Photoelectrodes for Photoeletrocatalysis

Lixia Gao, ^§1,2^ Jiale Xie, ^§1,2,3^ Xiaoqing Ma^1,2^ and Ling Yu^1,2^*

^1^ Institute for Clean energy & Advanced Materials, Faculty of Materials & Energy, Southwest University, Chongqing 400715, China

^2^ Chongqing Key Laboratory for Advanced Materials and Technologies of Clean Energies, Chongqing 400715, China

^3^ Institute of Materials Science and Devices, Suzhou University of Science and Technology, Suzhou 215011, China

*****Correspondence: Ling Yu, E-mail: lingyu12@swu.edu.cn; Tel.: +86-23-6825-4842

§ **Contribute equally to this work**

**First author: Lixia Gao, E-mail:** **gaolx@email.swu.edu.cn; Jiale Xie, E-mail: xiejiale@swu.edu.cn**

**Supplementary Materials:**


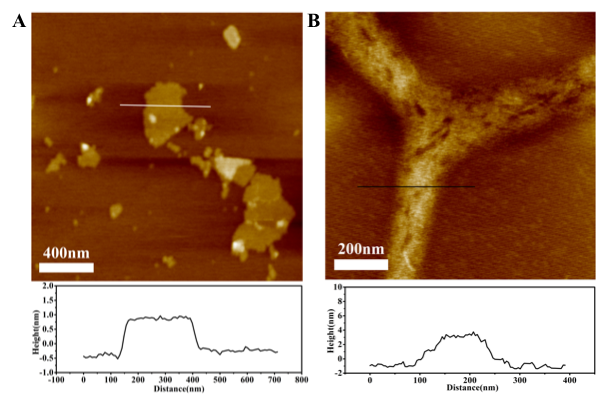


**Figure S1.** AFM images of (A) GO drop-casted on freshly cleaved mica surface, (B) DNA drop-casted on freshly cleaved mica surface and

**

**

**Figure S2.** XRD pattern of Mn_3_(PO_4_)_2_ powders. A. Mn_3_(PO_4_)_2_; B. standard XRD pattern of Mn_3_(PO_4_)_2_ from the PDF card of 33-0901.


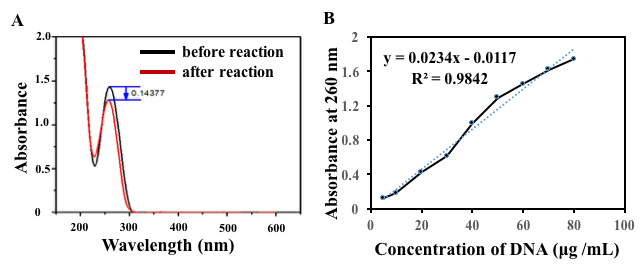


**Figure S3.** Absorbance of DNA solution before and after react with MnSO4 and K3PO4 (A); concentration vs absorbance calibration of DNA solution (B).


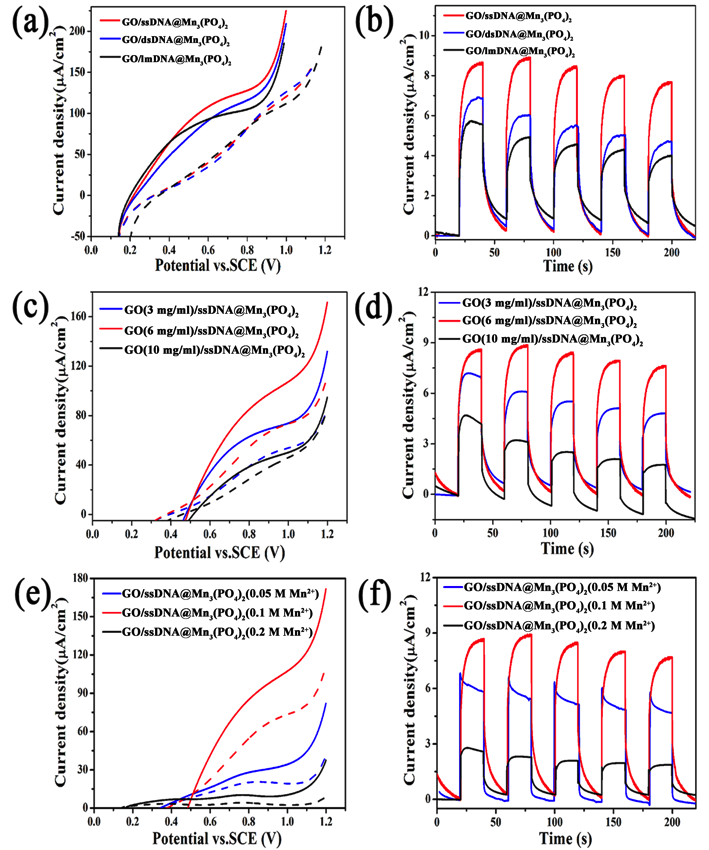


**Figure S4.** Linear sweep voltammetry curves of (a) ssDNA, dsDNA and lmDNA synthesized nanocomposite (c) different concentrations of GO synthesized nanocomposite (e) different concentration of Mn_3_(PO_4_)_2_; and photocurrent response of (b) ssDNA, dsDNA and lmDNA synthesized nanocomposite (d) different concentrations of GO synthesized nanocomposite (f) different concentrations of Mn_3_(PO_4_)_2_.

**
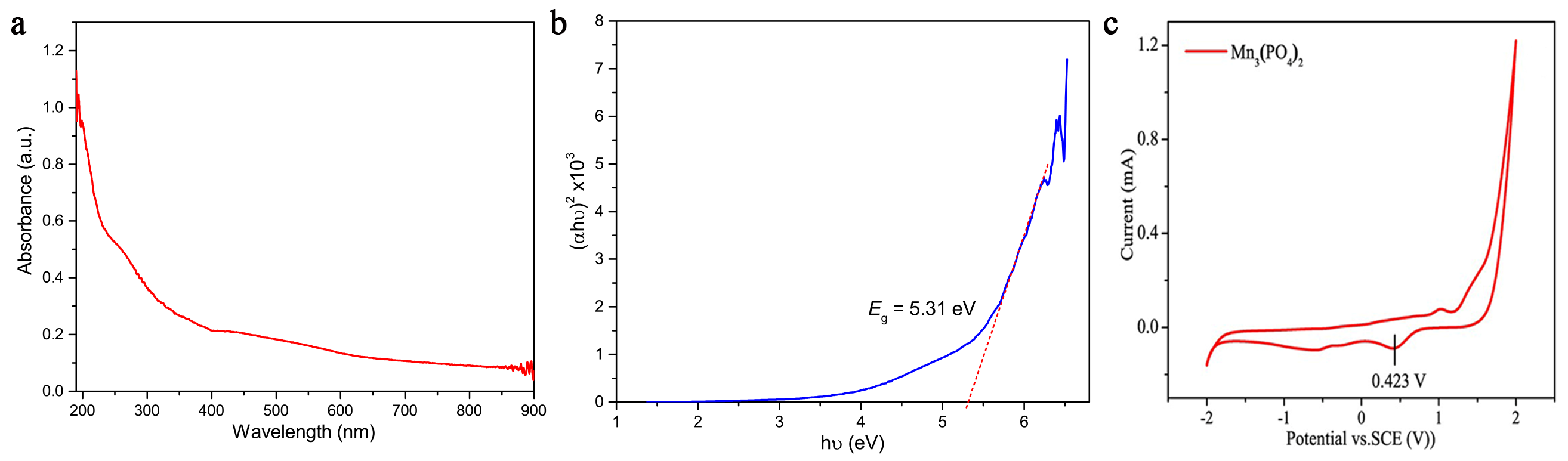
**

**Figure S5.** (a) UV-*vis* diffuses reflectance spectra of Mn_3_(PO_4_)_2_ powder, (b) first derivative absorption spectra of Mn_3_(PO_4_)_2_ powder. (c) Cyclic voltammetry (CV) curve of Mn_3_(PO_4_)_2_ on glassy carbon electrode in 0.1 M KCl solution.
